# Welfare of extensively managed Swedish Gotland ponies

**DOI:** 10.1017/awf.2023.20

**Published:** 2023-02-23

**Authors:** Sofie M Viksten, Elke Hartmann, Karin Schneller, Margareta Steen

**Affiliations:** 1 Hästfokus AB, Vickeby 84, 74190 Knivsta, Sweden; 2 Swedish University of Agricultural Sciences, Department of Animal Environment and Health, Box 7068, 750 07 Uppsala, Sweden; 3 Österängsgatan 24B, 753 28 Uppsala, Sweden; 4 Swedish University of Agricultural Sciences, Swedish Centre for Animal Welfare, Box 7053, 750 07 Uppsala, Sweden

**Keywords:** animal welfare, equine behaviour, extensive horse management, grazing, horse, welfare assessment

## Abstract

It has been suggested that grazing horses could be used as a credible tool for landscape conservation which would, at the same time, improve horse welfare as opposed to conventional housing. A study was conducted between May 2014 and April 2015 on 12 one year old Gotland ponies managed extensively without supplementary feed. Monthly animal welfare assessments (n = 13) revealed welfare issues in most of the horses, i.e. low body condition score (BCS < 3/5), recurring poor skin condition in 11/12 horses and ocular discharge in 7/12 horses. At the end of the study, compared to the beginning, chafing and poor skin condition increased while coat condition improved. A correlation was found between a negative reaction (score > 0) in the human approach test and BCS < 3 and ocular discharge. Avoidance Distance test values were correlated with faecal parasite counts (> 350 eggs per gram [EPG]). These results indicate that the horses had acceptable welfare during late spring/summer (May–September) and that some horses required additional feed during winter. The animal welfare protocol proved to be an efficient tool for monitoring welfare. The results showed that factors important for extensive management are: daily monitoring; enclosures that provide sufficient feed; access to recovery enclosure; and habituation of horses to human approach.

## Introduction

A reduction in the number of farms keeping livestock has resulted in a decrease in natural pasture in Sweden (Sandström *et al*. 2015). Since these pastures are often habitats for red-listed wildlife species, their decrease constitutes a serious threat to biodiversity (Gustavsson 2007; Sandström *et al*. 2015; Cousins *et al*. 2015). In order to address this issue, Swedish farmers may gain subsidies from the Swedish Rural Development Programme (Swedish Board of Agriculture [SBA] 2018b) for restoration, preservation and enhancement of such ecosystems. Grazing by large herbivores such as cattle and horses has positive effects on plant biodiversity, accommodation of fauna, ecosystem functioning due to selective browsing and grazing, and is seen as an efficient tool in grassland management and landscape conservation. Horses are very adaptable to a range of climates and their grazing provides additional positive effects compared with mowing (Slivinska & Kopij 2011; Tälle *et al*. 2016). They are therefore often regarded as key elements within landscape conservation programmes in different parts of the world, where the aim is restoration of ecological functions (Naundrup & Svenning 2015). Indigenous livestock breeds are preferable for use as grazers on otherwise abandoned grassland or wood-pastures since these breeds are thought to be better adapted to harsh environments. Moreover, these breeds are often categorised as endangered, whereby bringing them into useful service may secure their survival. Examples of such management programmes can be found in Belgium (Hoffmann 2002) and The Netherlands (Piek 1998).

The Gotland pony, or ‘Gotlandsruss’, is the only breed of horse indigenous to Sweden. It originally roamed freely on the island of Gotland in the Baltic Sea, possibly from the Iron Age until the early 19th Century. Numbers then decreased drastically due to agricultural intensification as well as ponies being sold to work abroad in mines. The breed was saved from extinction by the founding of the Gotlandsruss Breeding Association (Svenska Russavelsföreningen), which facilitated the establishment of an enclosure of approximately 600 hectares on Lojsta moor (Gotland) where the ponies continue to live in a semi-feral state (Graaf 2015). They are provided with supplementary forage during the winter months but are otherwise left undisturbed, except for annual hoof care, registration and removal of foals. The feral history of the breed, together with its environmental adaptations (thick winter coat and low energy requirement), make it an ideal candidate for use in grassland management and landscape conservation under free-ranging conditions. Extensive management regimes, however, may also lead to animal welfare concerns, e.g. exposure to extreme weather, thermal discomfort, health issues and even predation (Górecka-Bruzda *et al*. 2020). Horses may also become more fearful of humans if not handled regularly and may therefore show stress responses during unavoidable handling (Jezierski *et al*. 1999). Thus, under semi-feral conditions, it is of paramount (and possibly specific) importance to monitor the welfare status of each individual in the group in order to identify welfare issues and related risk factors to enable early interventions that safeguard their welfare (Blokhuis *et al*. 2010). Animal welfare may be defined as the inherent attempt of an individual animal to cope with its environment, including how it feels and how it experiences its situation (Bracke *et al*. 1999; Keeling *et al*. 2011). According to Fraser *et al*. (1997), farmed animals should be allowed to express their full behavioural repertoire in an environment appropriate to their physiology. Three levels of animal welfare have been identified, relating to Fraser (2003): biological functioning of the animal in terms of health, growth and productivity; ‘affective states’ of the animal concerning pain, suffering, feelings and emotions; and freedom for the animal to live under natural circumstances and express its normal behaviour.

In the domestic environment, horses may be prevented from expressing their behavioural repertoire because of environmental constraints, such as stabling in stalls, limited turn-out in small paddocks, often coinciding with low forage feeding, social isolation and lack of unrestrained exercise, all of which give rise to welfare concerns (Visser & Van Wijk-Jansen 2012). These concerns have led to an increased demand for keeping horses in 24-h loose-housing systems, where extensive, semi-feral keeping of horses is discussed as an alternative. Managing domestic horses on natural pastures year-around may benefit overall welfare, as it best fulfils their natural needs such as grazing and browsing, free movement and social contact with conspecifics (Viksten 2016).

Sweden has high horse welfare standards, established by the Animal Welfare Act (SBA 2018a), the Animal Welfare Ordinance (SBA 2019a), and the Swedish Board of Agriculture regulations on the care and management of horses (L101) (SBA 2019b), all presented in exceptional detail. The legislation aims to prevent negative welfare issues by stating minimal levels of management. One such requirement is to provide horses with additional feed during the winter months, enabling them to maintain healthy body condition (L101 Ch 4 §3–4;SBA 2019b; SBA 2018a Ch 2, §4). However, existing subsidies for keeping livestock, including horses, on semi-natural grassland of particular biological value, do not always permit provision of supplementary feed (SBA 2018b). This is to prevent sensitive plant species from being subjected to added nutrients and competition. Therefore, animal welfare legislation and the regulation for rural development oppose each other, which may set limits for using horses in grassland management. If it is possible to maintain welfare without supplementary feed, as will be explored in this study, this could lead to legislative amendments.

In order to safeguard horse welfare, regular monitoring is essential which requires protocols that measure all aspects of welfare, i.e. the animal’s perception of its environment, and includes changes over time together with presenting information related to management procedures and available resources (Mellor 2016). Several horse welfare assessment protocols exist, that apply mainly to horses kept and managed at a stable or indoor facility (Dalla Costa *et al*. 2016; Viksten *et al*. 2017; Czycholl *et al*. 2019; Hausberger *et al*. 2020), as opposed to extensively managed or free-ranging populations (Harvey *et al*. 2020). Examples of available horse welfare assessment protocols include: the Australian Horse Welfare Protocol (AHIC 2011), the Wageningen Assessment Protocol for Horses (Wageningen UR Livestock Research 2012), the Assessment Protocol for Horses (AWIN 2015) and the Horse Welfare Assessment Protocol (HWAP; Viksten *et al*. 2017). All these protocols share a focus on animal-based measures (e.g. observed behaviours, physical condition, injuries, early signs of disease) that reflect the welfare state of an individual (Dalla Costa *et al*. 2014; Lesimple 2020). Resource-based measures (e.g. availability and cleanliness of water, housing type, feeding regime) or management-based measures (e.g. time kept in paddock, group size) alone would be insufficient for a holistic assessment of equine welfare (Visser *et al*. 2014).

The main aim of the present study was to use an adapted version of the HWAP (Viksten 2016; Viksten *et al*. 2017), to monitor the welfare of a group of extensively managed Gotland ponies. It was hypothesised that the horses would cope well and experience good welfare throughout the year without supplementary feeding. A further aim was to identify relevant welfare parameters for monitoring horses in extensive management systems.

## Materials and methods

This project was approved by the Ethic Committee on Animal Experimentation in Uppsala, Sweden, under the protocol C28/14, and conformed with the guidelines for the ethical treatment of animals in applied animal behaviour research (Sherwin *et al*. 2003).

### Project outline and study area

The study was part of a larger project entitled ‘The Gotland pony as a conservationist: A way to promote the biodiversity and to conserve an endangered breed (2014–2016).’ Other studies within the larger project have focused on the impact of year-round grazing on pasture-nutrient dynamics in relation to faecal nutrient composition (Ringmark *et al*. 2019), parasite occurrence in Gotland ponies (Tydén *et al*. 2019), effects of year-round grazing on plant biodiversity, grassland functional composition and pollinator habitat use (Garrido *et al*. 2019).

The aim of the current study was to monitor the welfare of extensively managed Gotland ponies for one year, between May 2014 and April 2015 on the property of the University of Agricultural Sciences (SLU) in Krusenberg, south of Uppsala, Sweden (59°44’8”N, 17°38’58”E). Uppsala county is characterised by a humid, continental climate with warm summers and cold winters, although the latter are not as cold as other locations at similar latitudes, due to the warming effects of the Gulf Stream.

### Horses and management

Twelve one year old native Gotland pony stallions with a mean (± SD) bodyweight of 185 (± 21) kg at the start of the study, were purchased from six different breeders by SLU. The horses spent the first month together in an enclosure of approximately three hectares, before being split into three groups of four individuals and randomly allocated to an enclosure in May 2014. Horses purchased from the same breeder with the same sire were allocated to different groups. Group allocation was also based on coat colour, i.e. horses with distinct colours were placed in the same group for easier identification (seven bay horses and five chestnuts, of which one had a flaxen mane). The horses were relatively unhandled prior to the start of the larger study and were therefore all handled and trained to be haltered and led.

The three enclosures were of similar size (10–13 hectares) and consisted of one-third grassland and two-thirds forest, based on calculated estimates of grass production to meet the horses’ energy requirements (Ringmark *et al*. 2019). Detailed descriptions of vegetation types in each enclosure are presented in Ringmark *et al*. (2019) and Garrido *et al*. (2019, 2021). A roofed, three-walled shelter (Mobile Covers, Cover all Europe GmbH, Groß Lüdershagen, Germany) measuring 16 m^2^ (height 3.15 m), was set up in all enclosures (for placement, see Ringmark *et al*. 2019) as required by legislation. In each enclosure, water was provided *ad libitum* during the warm season from two automatic water-troughs and one manual trough. When the temperatures dropped below 0°C, water was offered manually in plastic troughs once per day. The horses did not receive any supplementary feed except salt blocks with trace minerals. However, if a horse’s body condition score (BCS) fell below 2 on a scale from 0 to 5 (Carroll & Huntington 1988) during the cold season, it was transferred to a recovery enclosure (with a shelter), covered with a rug and given supplementary feed until BCS normalised.

Besides HWAP assessments (Viksten *et al*. 2017) at regular four-week intervals (see below), the horses were also monitored daily by staff from SLU for health issues (e.g. general condition and lameness) indicative of reduced welfare (Tydén *et al*. 2019). Moreover the horses remained under veterinary supervision for the duration of the study.

### Data collection

Horse welfare was assessed every fourth week from May 2014 until April 2015, giving a total of 13 assessments for each individual and a total of 156 individual observations, using a selection of measures considered relevant for extensive horse management as per the HWAP protocol (Viksten *et al*. 2017). Measures related to stables, training and feeding regimes were excluded. At each monthly assessment, animal- and resource-based measures were recorded. The animal-based measures included a behavioural assessment using the Human Approach (HA) and Avoidance Distance (AD) test, which were carried out before proceeding with the recording of physical health measures and resource-based measures.

In the HA test (Burn *et al*. 2010; Popescu & Diugan 2013), the assessor walked calmly towards the horses without adopting any specific body posture. The horses were always aware of the assessor’s approach and recordings started when the person arrived at a distance of at least 5 m from the group. The horses’ reactions were scored individually according to Table 1, starting with the horse closest to the assessor.

**Table 1.** Animal-based behavioural measures adapted from Popescu and Diugan (2013) and Burn *et al*. (2010), used in the Human Approach and Avoidance Distance tests on horses

Parameter	Score	Description
Human Approach test	0	Positive, the horse moves towards or turns its head towards assessor
	1	Neutral, no distinct movement towards assessor, may include turning ears towards assessor without turning the head
	2	Negative, aggression with ears flattened, may include threat to bite or turning hindquarters towards assessor or avoidance by moving away
Avoidance Distance test	0	Possible to touch the horse’s muzzle with outstretched hand
	1	Closer than 0.5 m but unable to touch
	2	Between 0.5 m and 1 m from muzzle with outstretched hand before the horse turns away
	3	Not possible to come closer than 1 m from muzzle with outstretched hand

The AD test (Popescu & Diugan 2013) was carried out immediately after the HA test. The assessor walked calmly towards the horse that was closest, stretching out a hand at an angle of about 45º. The horse’s initial reaction to the approach was scored according to Table 1. After this first assessment of the horse closest to the person, the remaining three horses in the group were approached one-by-one, always starting with the individual nearest the assessor.

The physical health assessment (see Table 2) was carried out by both visual inspection of the horse’s whole body, and palpation. If the horse did not stand still, it was haltered, and if it could not be haltered it was assessed visually. Scores were on a scale from 0 to 2 where 0 reflected the least severe and 2 the most severe health condition. Some measures were binary, i.e. 0 = not present and 1 = present. Body condition was evaluated by palpating the neck, withers, back and loin, ribs and hindquarters from both sides as described by Wright *et al*. (1998), using a combination of the systems presented by Carroll and Huntington (1988) and Henneke *et al*. (1983). The BCS scale (Table 3) ranged from zero (very thin) to five (obese) and included half points.

**Table 2.** Animal-based measures used to assess horse physical health in accordance with the Horse Welfare Assessment Protocol (HWAP; Viksten *et al*. 2017)

Parameter	Score	Description
Respiration	0	No abnormal flank movements
	1	Abnormal flank movements (deep or quick)
Thermal comfort	0	No sweating or shivering
	1	Sweating or shivering
Hoof condition	0	Normal, no indication of hoof problems
	1	Some cracks or long hooves
	2	Severe cracks and abnormal shape, overgrown
Wounds	0	No wounds
	1	Wounds with hair loss, no skin perforation
	2	Wounds < 3 cm skin perforation
	3	Wounds > 3 cm
Chafing	0	No indication of chafing
	1	Indication of chafing (hairless spots, broken hairs)
Skin condition	0	Normal skin condition without problems
	1	Skin problems such as dandruff, crusts, dermatitis, sunburn, insect bites on a few spots
	2	Large parts of the skin affected
Coat condition^1^	0	Sleek, glossy coat
	1	Dull, dry coat, partially long
Mane and tail condition	0	Normal with no chafes or broken hair
	1	Chafes in mane/tail with hairless spots, broken hairs < 10 cm in mane, 5×5 cm in tail
	2	Severe signs of chafing with perforated skin
Ocular discharge	0	No ocular discharge
	1	Dirty eye with mucus in the corner of the eye
	2	Dirty eye with or without mucus in the corner of the eye or all around it, and eye discharge
Nasal discharge	0	No presence of nasal discharge or uncoloured discharge
	1	Presence of coloured or thick nasal discharge in at least one nostril
Lameness^2^	0	No visible asymmetry or abnormal movement
	1	Visible asymmetrical movement
	2	Lame with no weight-bearing on one leg

1 Season was taken into consideration so that winter coat was not assessed as long.

2 Lameness was assessed while the horse was moving, usually in walk.

**Table 3.** Body condition scoring performed as part of horse physical health assessment. Fat deposits on the neck, withers, back and loin, ribs and hind quarters were assessed by palpation according to Wright *et al*. (1998)

Score	Description	Area	Assessment
0	Very thin/emaciated	Neck	Bone structure easily felt- no muscle shelf where neck meets shoulder
		Withers	Bone structure easily felt
		Back and loin	Vertebrae points easily felt
		Ribs	Each rib easily felt
		Hindquarters	Tail-head and hip bones projecting, sunken rump, deep cavity under tail
1	Thin/poor	Neck	Bone structure can be felt, slight shelf where neck meets shoulder
		Withers	Bone structure can be felt
		Back and loin	Spinous process easily felt - transverse processes have slight fat covering
		Ribs	Slight fat covering, but ribs can still be felt, ribs visible
		Hindquarters	Hip bones can be felt, prominent pelvis
2	Fair/moderate	Neck	Fat covering over bone structure
		Withers	Fat deposits over withers, depending on conformation
		Back and loin	Fat over spinous processes
		Ribs	Ribs not visible, but can still be felt
		Hindquarters	Hip bones covered with fat,
2.5			e.g. racing condition or endurance horse
3	Good	Neck	Neck flows smoothly into shoulders
		Withers	Neck rounds out withers
		Back and loin	Back is level
		Ribs	Layer of fat over ribs
		Hindquarters	Hip bones cannot be felt, covered in fat and rounded,
3.5			e.g. mature mare mid-gestation
4	Fat	Neck	Fat deposited along neck
		Withers	Fat padded around withers
		Back and loin	Positive crease along back
		Ribs	Fat spongy over and between ribs, requires firm pressure to be felt
		Hindquarters	Hip bones cannot be felt
5	Very fat	Neck	Bulging fat
		Withers	Bulging fat
		Back and loin	Deep positive crease
		Ribs	Pockets of fat, ribs cannot be felt
		Hindquarters	Pockets of fat, bones cannot be felt

Evaluation of resource-based measures included visual assessment of water availability and cleanliness in the trough (Table 4).

**Table 4.** Resource-based measures used to assess the water cleanliness and availability according to the Horse Welfare Assessment Protocol (HWAP; Viksten *et al.* 2017)

Parameter	Score	Description	
Water access	0	Access to water
	1	No access to water
Water cleanliness	0	Water and trough clean
	1	Water or trough dirty/slimy
	2	Both water and trough dirty/slimy

The assessments were carried out by Masters students extensively trained by an experienced animal welfare assessor familiar with the methodology (Viksten 2016). Training included the study of relevant scientific literature, followed by practical training on around 40 horses of various breeds and body types. The training was considered complete when repeatability was ≥ 80% agreement with the supervisor and a golden standard. During assessments in the field, there were always two people present for safety reasons and if horses showed aggression or severe avoidance, physical assessment was not undertaken in order to minimise risk of injury. If possible, a visual assessment was carried out instead.

Parasite occurrence was monitored by Tydén *et al*. (2019), who describe in detail how egg counts for parasites were carried out through faecal sampling.

Meteorological data for the study period were obtained from the Swedish Meteorological and Hydrological Institute’s (SMHI) website (SMHI 2021).

### Statistical analysis

All statistical analyses were performed in the statistical software programme IBM SPSS^®^ version 25.

#### Comparison between start and end

The individual behavioural data and health status of the 12 horses at the start of the study (May 2014), were compared with the outcome at the end of the study 12 months later. The last two measurement dates (March and April 2015) were pooled to increase the statistical stability. Fisher’s exact test was applied to examine whether changes were statistically significant.

#### Seasonal variation in welfare parameters

Time series graphics were used for explorative analysis of the data, to assess variation of behavioural and health parameters over the study period. CHAID (Chi-squared automatic interaction detection using the decision tree technique to predict response variables) analysis based on Pearson Chi^2^ was used to assess whether the time of year had a significant effect on welfare parameters. Season was defined as spring (March–May), summer (June–August), autumn (September–November) and winter (December–February).

#### Relationships between animal welfare parameters

To identify relationships between welfare parameters, different analytical methods were used. CHAID analysis was applied to identify welfare parameters with a significant effect on selected dependent variables, such as BCS, health parameters and behavioural variables. Statistically significant results were split into subgroups based on Pearson Chi^2^ correlations. Graphical scatterplots were applied to visualise correlations and identify outliers for aggregated data at group level. Spearman rank correlation was used on aggregated data to explore correlations between welfare measures. Since the study was explorative in nature, due to the low number of horses, correction for multiple comparisons was not performed.

All data were analysed at group level. This data aggregation was carried out to reduce time fluctuations and unexplained variance partly caused by time series. Results are presented as number and percentage of horses, unless otherwise stated. Each horse in an enclosure was allocated a letter code (A–D) and a number representing the enclosure (1–3). The significance level was set at *P* < 0.05.

## Results

### Weather conditions

Winter 2014–2015 was considered mild, with average monthly temperatures exceeding normal averages by several degrees in Uppsala County (SMHI 2021) (Table 5). The snow cover was approximately 0–10 cm in December 2014 and 0–30 cm in January and February 2015 (Labartino *et al*. 2015; SMHI 2021).

**Table 5.** Average monthly temperature, humidity and precipitation measured and calculated for Uppsala County based on SMHI data (reviewed May 2021). Normal monthly temperature refers to observations 1961–1990

Month and year	May 2014	Jun 2014	Jul 2014	Aug 2014	Sep 2014	Oct 2014	Nov 2014	Dec 2014	Jan 2015	Feb 2015	Mar 2015	Apr 2015	May 2015
Average monthly temperature (°C)	10.9	13.7	20.5	16.9	12.5	8.5	4.3	–1.2	0.0	0.7	3.4	7.0	9.8
Normal monthly temperature (°C)	10.4	15.0	16.4	15.2	10.9	6.4	1.2	–2.6	–4.2	–4.3	–0.7	4.1	10.4
Average monthly precipitation (mm)	58	65	42	122	50	69	35	31	85	22	35	14	83
Normal monthly precipitation (mm)	33	45	75	65	59	50	52	43	38	27	28	29	33

### Comparison between start and end

At the start of the study, six horses (1B, 1C, 1D, 2C, 2D, 3B) had BCS < 3, three horses (1A, 2A, 2C) had ocular discharge and five horses (1C, 1D, 2C, 3B, 3C) had signs of chafing.

When comparing the data at the start of the study (May 2014) with data at the end (March–April 2015), a statistically significant increase in chafing score > 0 (*P* = 0.002) was seen, with chafing recorded in 42 and 92% of the horses, respectively (see Table 6). Poor skin condition increased significantly (*P* = 0.03), from 0 to 41% of horses that scored > 0 at the end of the study (Table 6). Coat condition improved (*P* < 0.001) from score 1 to score 0 in 88% of the horses (Table 6). No significant change was seen in behavioural scores in the AD and HA tests, respiration, thermal comfort, hoof condition, ocular discharge, nasal discharge, wounds, mane and tail condition or lameness, when comparing the beginning with the end of the study (Table 6).

**Table 6.** Comparison of behaviour and health status at the start of the study (May 2014) and at the end (March–April 2015), analysed using Fischer’s exact test with March and April pooled as a mean and analysed as one assessment

Measure	May	March	April	
	Score, n = 12	Score, n = 12	Score, n = 12	*P*-value
Human Approach test	0 = 12	0 = 12	0 = 12	*P* > 0.05
	1 = 0	1 = 0	1 = 0	
	2 = 0	2 = 0	2 = 0	
Avoidance Distance test	0 = 11	0 = 11	0 = 11	*P* = 0.27
	1 = 0	1 = 1	1 = 1	
	2 = 0	2 = 0	2 = 0	
	3 = 1	3 = 0	3 = 0	
Respiration	0 = 12	0 = 12	0 = 12	*P* > 0.05
	1 = 0	1 = 0	1 = 0	
Thermal comfort	0 = 12	0 = 12	0 = 12	*P* > 0.05
	1 = 0	1 = 0	1 = 0	
Hoof condition	0 = 12	0 = 12	0 = 12	*P* > 0.05
	1 = 0	1 = 0	1 = 0	
	2 = 0	2 = 0	2 = 0	
Wounds	0 = 8	0 = 11	0 = 10	*P* = 0.24
	1 = 3	1 = 1	1 = 2	
	2 = 1	2 = 0	2 = 0	
	3 = 0	3 = 0	3 = 0	
Chafing	0 = 7	0 = 1	0 = 1	*P* = 0.002
	1 = 5	1 = 11	1 = 11	
Skin condition	0 = 12	0 = 9	0 = 6	*P* = 0.03
	1 = 0	1 = 3	1 = 6	
	2 = 0	2 = 0	2 = 0	
Coat condition	0 = 1	0 = 11	0 = 10	*P* < 0.001
	1 = 11	1 = 1	1 = 2	
Mane and tail condition	0 = 12	0 = 12	0 = 12	*P* > 0.05
	1 = 0	1 = 0	1 = 0	
	2 = 0	2 = 0	2 = 0	
Ocular discharge	0 = 9	0 = 12	0 = 10	*P* = 0.13
	1 = 3	1 = 0	1 = 2	
	2 = 0	2 = 0	2 = 0	
Nasal discharge	0 = 12	0 = 12	0 = 12	*P* > 0.05
	1 = 0	1 = 0	1 = 0	

### Seasonal variation in animal welfare parameters

Time series analysis of the HA test results showed that on two occasions, July 2014 (n = 8) and November (n = 7), significantly (*P* < 0.001) more horses were difficult to approach (score > 0).

Season had a significant effect on BCS (*P* = 0.001). Horses with low BCS (< 3) at the onset of the study continued to have low BCS in other assessments: 1A in ten assessments, 1B in eight, 1C in seven, 2C and 3B in six and 2D in three. Horses 1A, 2A, 2B, 3A and 3D, with initial BCS ≥ 3, had periods with BCS < 3, and from December 2014 to February 2015 at least 73% of all horses had BCS ≤ 2.5. One individual, 3C, never scored < 3. Horse 3A scored > 0 (not possible to touch) in the AD test during six of the final seven assessments and, during 2015, only a visual assessment of BCS could be performed on that horse.

Horses 1B, 1C, 1D and 3B were moved to the recovery enclosure for 1–4 weeks in February–March 2015 to recover from low BCS. In the recovery enclosure, they were covered with a rug and given supplementary feed. The horses were returned to their original enclosure when BCS was restored (score > 2).

Chafing (score > 0) increased significantly (*P* = 0.03) between February and April 2015 in 92% of the horses. In the final assessment in April 2015, six horses scored > 0 on skin condition, eleven horses scored > 0 on chafing, and six of the horses (all bays) had ectoparasites of the species *Bovicola equi* (chewing lice).

**Figure 1.**
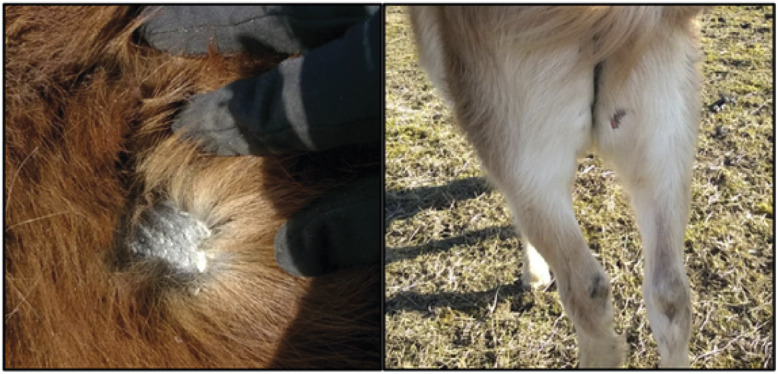
Skin condition (left) scored > 0 and wounds (right) scored 1 on the horses. Images courtesy of Labartino *et al*. (2015).

Skin condition deteriorated (score > 0) significantly (*P* < 0.03) over time and 92% of the horses scored > 0 at least once during the study (Figure 1). Compared with chestnut horses (n = 5), bay horses (n = 7) had skin condition scores > 0 for a significantly (*P* = 0.001) longer period.

In February 2015, three horses showed signs of diarrhoea.

### Relationships between animal welfare parameters

**Figure 2.**
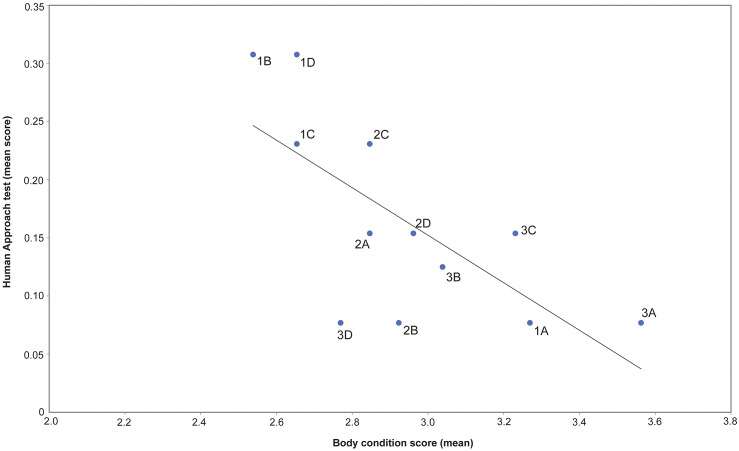
Correlation (Spearman rank correlation: *r* = –0.70; *P* = 0.01; see trendline) between mean individual scores in the Human Approach (HA) test and mean body condition score (BCS) where horses with BCS < 3 had an HA score of > 0. The values for individual horses are shown as labelled points.

There was a significant negative correlation between the HA test result and BCS (*r* = –0.70; *P* = 0.011), ocular discharge (*r* = 0.733; *P* = 0.007) and lameness (*r* = 0.727; *P* = 0.007), i.e. horses scoring > 0 for these parameters often scored > 0 in the HA test (Figure 2). Seven horses with ≥ 2 welfare issues (score > 0) had a score of > 0 in the HA test.

The AD test values showed a weak positive correlation (Spearman) with occurrence of wounds (*r* = 0.217; *P* = 0.009) and a negative correlation with absence of chafing (*r* = –0.164; *P* = 0.044).

There was a significant correlation between AD test values and parasites/egg per gram (EPG) counts (*r* = 0.761; *P* = 0.004). Three horses with > 350 EPG (yearly average) had a statistically significant correlation with AD test scores > 0. Nine horses with < 350 EPG scored 0 in the AD test.

Chafing and coat condition had a significant (Pearson Chi^2^ *P* = 0.049) correlation with BCS. In 46 individual observations, horses with no chafing (score 0) and good coat condition (score 0) had significantly higher BCS than horses with no chafing (score 0) and poor coat condition (score 1) observed in 17 individual assessments.

Eight of the 12 horses (1A, 1C, 1D, 2B, 2C, 3B, 3C, 3D) had wounds (score 1 in 67% of horses, score 2 in one horse), two horses (1B, 1D) showed lameness (score 1) at one and two assessments, respectively, and seven horses (1B, 2A, 2B, 2C, 2D, 3B, 3D) had some hoof cracks (score 1) in one or a few assessments. However, none of these results were statistically significant.

**Figure 3.**
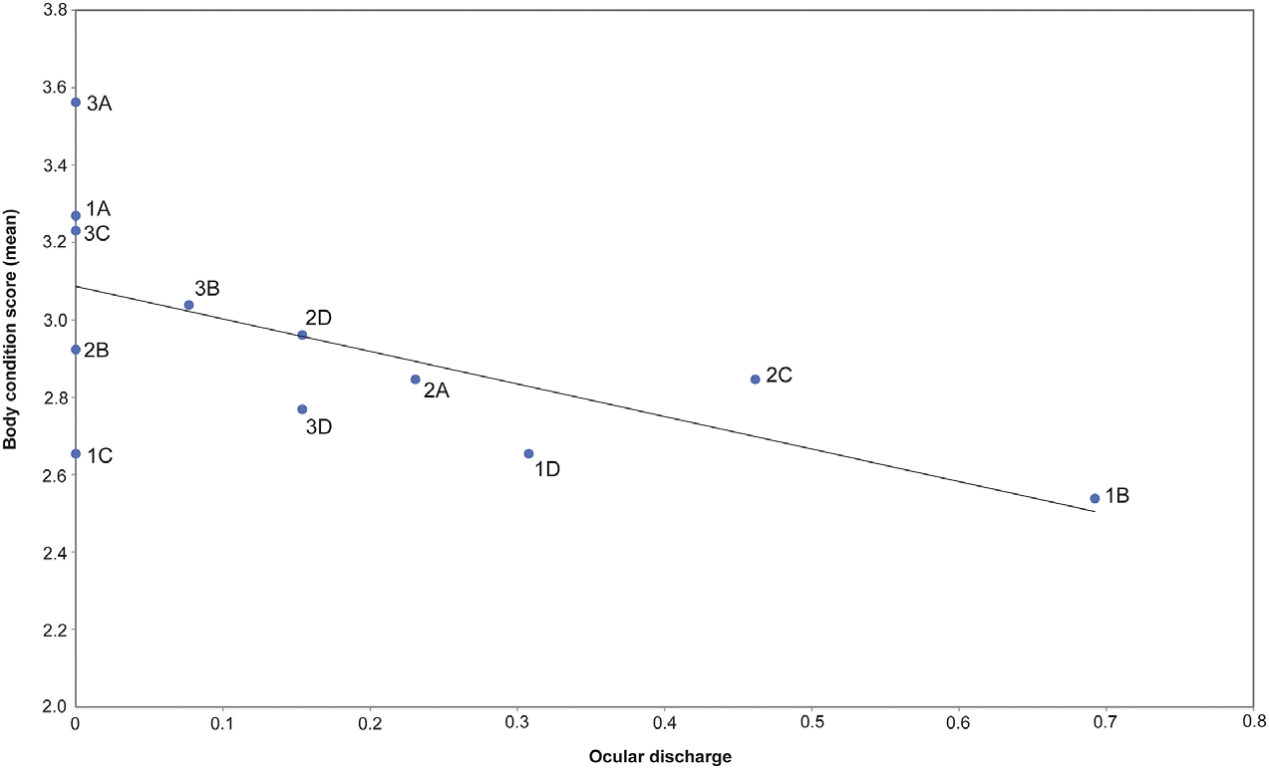
Correlation between body condition score (BCS) and presence of ocular discharge (Spearman correlation: *r* = –0.63; *P* = 0.03). Horses with higher BCS had no ocular discharge, while horses with lower BCS had ocular discharge. Ocular discharge is presented as average value per horse for the study period. The values for individual horses are shown as labelled points.

A correlation was seen between ocular discharge (score > 0) and BCS < 3. Six horses with BCS < 3 had an ocular discharge score of 1. Two horses with BCS < 3 and three horses with BCS > 3 did not have ocular discharge (score 0) (Figure 3).

**Figure 4.**
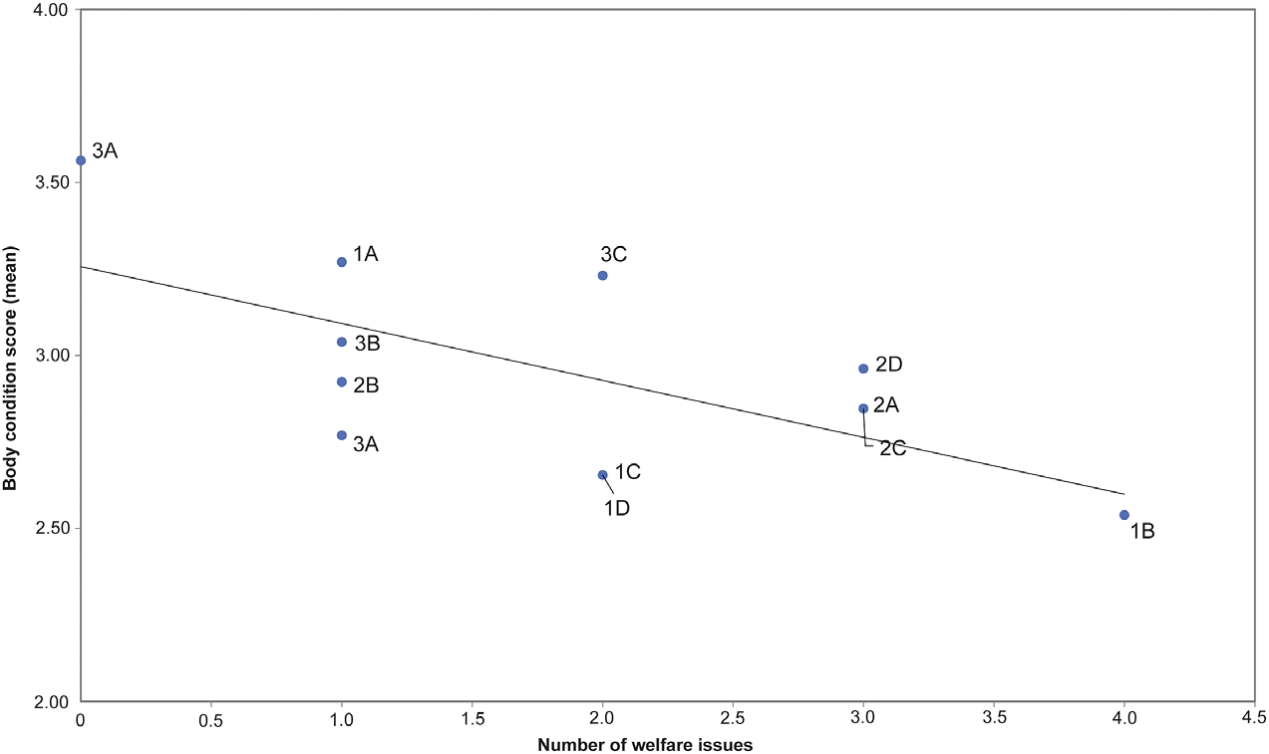
Correlation between number of welfare parameters assessed (presented as average per horse for the study period) with score > 0 (Pearson correlation: *r* = –0.644; *P* = 0.024) and body condition score (BCS) based on aggregated data from all individuals: the more welfare issues the lower the BCS. The values for individual horses are shown as labelled points.

There was a correlation between the number of welfare issues assessed (score > 0) and BCS < 3, when analysing aggregated data for all individuals (Figure 4). Almost all horses (11/12) had welfare issues at some point during the study, up to seven (often mild) issues per assessment (out of 13). When analysing the average number of welfare issues per horse for the study period, eleven out of 12 horses had issues; one horse (3A) had no welfare issues, four (1A, 2B, 3A, 3B) had one issue, three horses (1C, 1D, 3C) had two issues, three horses (2A, 2C, 2D) had three issues and one (1B) had four issues (Figure 4).

## Discussion

The Uppsala region in central Sweden can experience winters with heavy snowfall and temperatures well below zero. Such conditions will increase the energy requirements of horses and the need for supplementary feed and shelter, which are requirements according to the Swedish horse welfare legislation (SBA 2019b) for horses kept permanently outdoors. Winter 2014–2015 was considered mild with less than average snow cover (barely exceeding 30 cm on a few occasions), and thus good grazing availability. Nevertheless, welfare assessments revealed that several horses required supplementary feed due to low BCS and eleven of the horses had (often mild) welfare issues (score > 0) at one or more assessments. Although this varied with season, horses with multiple issues had significantly lower BCS and were more reluctant to be approached in the HA test than horses with fewer welfare issues.

Horses kept extensively are expected to cope well even in sub-zero temperatures, if they have access to sufficient feed, shelter, and water (Brinkmann *et al*. 2013; Mejdell *et al*. 2020). All enclosures in this study were estimated to contain sufficient feed for the horses, yet most horses struggled to maintain good BCS. During the study, four horses had to be moved to the recovery enclosure during the cold season and given supplementary feed due to low BCS. Previous studies have shown that enclosure selection may prove the greatest challenge in extensive management. Thus, Saastamoinen *et al*. (2017) and Fleurance *et al*. (2010) suggest optimising the number of horses in an enclosure to safeguard welfare, but calculation of feed requirements and the amount produced in enclosures remain a challenge. This underlines the need for continuous welfare monitoring since even a hardy breed, such as the Gotland pony, may require supplementary feed in order to maintain acceptable welfare in winter and springtime, as reported previously (Brinkmann *et al*. 2013).

Horse body condition is affected by other factors in addition to availability and quality of feed, such as the general state of welfare (Christie *et al*. 2006) and drinking preferences (Houpt *et al*. 2000). Moreover, studies of feral horses have shown a negative correlation between parasite pressure and BCS (Debeffe *et al*. 2016). In February 2015, when BCS was low in most horses in this study, three horses showed signs of diarrhoea. This coincided with the occurrence of *Oxuyris equi* (pinworm) and *Parascaris* spp as observed by Tydén *et al*. (2019), requiring double treatments with deworming drugs (Tydén *et al*. 2019).

Assessments by Labartino (2015) showed that when the snow depth exceeded 20 cm and the average temperature was 1°C, the horses spent nearly 80% of their time sheltered in the forest, browsing and gnawing bark. Once the snow thawed and the temperature rose, the horses started to roam more widely within the enclosures and feeding activity increased along with BCS. A similar foraging observation, and seasonal fluctuations in general welfare, were also observed by Hampson *et al*. (2011), Boyd and Houpt (1994) and Brinkmann *et al*. (2013). Four horses (1B, 1C, 1D, 3B) had to be moved to an adjacent recovery enclosure during some parts of the winter, to recuperate from either low BCS or for monitoring of lameness. The reduced BCS during winter may also have been due to growth rate; young stallions have high energy requirements and these might not have been met during periods of the study. One horse out of the 12 never scored below 3 on BCS which indicates that some individuals may be more suited to extensive keeping without supplementary feed than others. It is also worth considering that the study did not have a natural herd composition, as one year old stallions would usually remain within their family group, which may have affected foraging behaviour and thereby the results (Morel *et al*. 2006). Moreover, it would have been an advantage to choose horses that are as similar as possible regarding, e.g. BCS at the onset. Starting differences in BCS, as seen in this study, may influence the outcome of BCS scoring and the evaluation of related welfare parameters.

An animal welfare issue that affected most horses during the study was recurring poor skin condition to varying degrees, which increased significantly over time. This might have been related to parasite burden or malnutrition (Scott & Miller 2010). Signs of chafing also increased significantly and, between February and April 2015, eleven of the 12 horses displayed chafing. Horses that did not chafe and had good coat condition, had higher BCS than horses that did not chafe but had poor coat condition. This is most likely due to the importance of coat quality for maintaining thermal balance during the colder months. Thus, scoring coat quality may be useful when assessing horse welfare under semi-feral conditions, since it might predict a lowered welfare state and a need for intervention.

Ectoparasites such as *B. equi* (chewing lice) were observed in six horses and coincided with signs of chafing and poor skin condition in the final assessments. Another possible reason for the observed skin problems, apart from ectoparasites, might be that the horses spent most of their time foraging, which meant greater exposure to the elements during the mild winter. When horses are subjected to wet, cold conditions and unable to dry their coats, skin problems such as dandruff, small wounds, itching and scabs may appear (Scott & Miller 2010). Poor skin condition may also lead to more serious welfare issues if left untreated, thereby affecting horse behaviour and energy intake. Coat condition improved at the end of the study compared with the beginning, most likely due to the foal coat being shed and replaced by the longer and glossier coat typically associated with adult horses.

Horse behaviour assessed during HA tests was affected by welfare issues recorded during the monthly assessments, i.e. horses with low BCS and ocular discharge were reluctant to be approached at two assessments (in July and November). In July, a significant increase in BCS occurred and, in November, a significant decrease in BCS was observed: the horses may have been focused on foraging and maybe not interested in human contact (since this was not associated with food). The exact reasons behind this reluctance for human approach requires further studies.

The AD test showed a weak correlation between occurrence of wounds and reluctance to be approached. Horses that did not show signs of chafing were easier to approach. An interesting finding was the strong correlation between avoidance of contact and faecal parasite counts, where horses with more than 350 EPG avoided human approach. This behaviour might have been due to associating human contact with the administration of oral deworming medication (Tydén *et al*. 2019), which was applied several times during the study. One horse avoided contact in six of the final seven assessments, meaning its welfare could only be monitored visually, which is ineffective since most parameters require physical examination of the horse. Thus, horses kept under this type of managerial regime need to be trained to accept approach, to enable palpation and sufficient welfare monitoring. Hence, the findings of this study demonstrate the importance of including behavioural assessments, particularly avoidance behaviour during human approach, as this can reflect the welfare status of the horse and is an important tool for animal welfare monitoring (Lesimple 2020).

Ocular discharge was detected throughout the study and did not change significantly between the beginning and the end of the study. Horses with ocular discharge tended to have lower BCS, which indicates that ocular discharge is suitable as an early indicator of reduced welfare, as supported by other studies (Dalla Costa *et al*. 2014).

Mild lameness was observed in two horses and reoccurred in one, which was therefore moved to the recovery enclosure and examined by a veterinarian. Lameness is a welfare concern due to its association with pain and impact on behaviours, such as foraging or inability to keep up and interact with conspecifics (Harvey *et al*. 2020). Therefore, an effective lameness assessment is very important and should be incorporated into all protocols as an indicator of impaired welfare.

The horses in this pilot study were able to express their natural social behaviours and fulfil their need to graze and move freely, which are important aspects of horse welfare (SBA 2016). However, the results showed that extensive horse management poses several challenges regarding animal welfare. The Swedish Board of Agriculture regulations on the care and management of horses (L101) (SBA 2019b) state that horses should be fed in accordance with their energy and nutritional needs, so that they maintain healthy body condition over time. This may pose a challenge in extensive management systems, as observed in this study.

The monitoring results revealed that some horses had up to seven welfare issues (score > 0) per assessment. Although this varied throughout the study, the horses in question had significantly lower BCS and were more reluctant to be approached in the HA test than horses with fewer welfare issues. This shows that several less severe welfare issues combined had a negative impact on overall welfare and that behavioural tests are an important part of welfare monitoring. The horses might have fared better had they been supplied with *ad libitum* roughage throughout the winter, as was concluded in a study of Shetland ponies, another robust breed, kept in outdoor housing with additional feed supply (Brinkmann *et al*. 2013). However, this is not feasible on many grasslands in need of conservation and grazing, due to fear of introducing invasive plant species and addition of nutrients detrimental to sensitive species. One way to achieve restoration of sensitive grassland and meet the requirements of good horse welfare, without introducing nitrogen and foreign invasive seeds, could be to use horses for grazing during the warmer months (late spring–autumn) and keep them elsewhere with supplementary feed in winter.

This study showed that a hardy breed such as the Gotland pony can manage on extensive pastures without supplementary feed with acceptable welfare during late spring and summer, but not during late autumn and winter. However, there was individual variance and some of the horses managed better than others year round.

Further studies on more horses of different breeds and ages, over a longer time-period, are required to confirm the welfare effects of managing horses extensively without supplementary feed under Nordic conditions.

### Animal welfare implications

This is the first study, to our knowledge, to use an established welfare monitoring protocol (HWAP; Viksten *et al*. 2017) to assess the welfare of extensively managed horses under Nordic conditions. The results show that early signs of reduced welfare can be reliably recorded, thereby allowing early interventions that safeguard horse welfare. The results can be used to raise awareness and increase understanding of animal welfare challenges, that can potentially arise when horses are kept under semi-feral conditions without supplemenary feed under Nordic conditions.

Based on the results obtained, the following recommendations for animal welfare monitoring of extensively kept horses in Sweden can be made:

- Ideally, all horses need to be trained to permit approach, in order to allow sufficient assessment of their physical health;
- A full animal welfare assessment according to an existing protocol, and conducted by a trained assessor, is required at regular intervals: at least once monthly;
- Animal-welfare assessment protocols should include behavioural measures of welfare, such as Avoidance Distance and Human Approach tests;
- Parasite monitoring might be required more often than in other management systems;
- Enclosures need to provide sufficient and surplus feed for all horses, free water access, and adequate shelter;
- An adjacent recovery enclosure should be established;
- Extensive management without supplementary feed can be suitable for spring and summer, but may not apply to late autumn and winter.

## References

[r1] Australian Horse Industry Council (AHIC) 2011 Australian Horse Welfare Protocol. AHIC: Geelong, Victoria, Australia.

[r2] AWIN 2015 *AWIN welfare assessment protocol for horses.* 10.13130/AWIN_HORSES_2015

[r3] Blokhuis H, Veissier I, Miele M and Jones B 2010 The Welfare Quality® project and beyond: Safeguarding farm animal well-being. Acta Agriculturae Scand Section A60: 129–140.

[r4] Boyd L and Houpt KA 1994 Przewalski’s Horse: The History and Biology of an Endangered Species. State University New York Press: Albany, NY, USA.

[r5] Bracke M, Spruijt B and Metz J 1999 Overall animal welfare assessment reviewed. Part 1: Is it possible? Njas-Wageningen Journal of Life Sciences 47: 279–291.

[r6] Brinkmann L, Gerken M and Riek A 2013 Effect of long-term feed restriction on the health status and welfare of a robust horse breed, the Shetland pony (*Equus ferus caballus*). Research in Veterinary Science 94: 826–831. 23141417 10.1016/j.rvsc.2012.10.010

[r7] Burn CC, Dennison TL and Whay HR 2010 Relationships between behaviour and health in working horses, donkeys, and mules in developing countries. Applied Animal Behaviour Science 126: 109–118.

[r8] Carroll C and Huntington P 1988 Body condition scoring and weight estimation of horses. Equine Veterinary Journal 20: 41–45. 3366105 10.1111/j.2042-3306.1988.tb01451.x

[r9] Christie JL, Hewson CJ, Riley CB, McNiven MA, Dohoo IR and Bate LA 2006 Management factors affecting stereotypies and body condition score in nonracing horses in Prince Edward Island. The Canadian Veterinary Journal 47: 136–143. 16579039 PMC1345728

[r10] Cousins SA, Auffret AG, Lindgren J and Tränk L 2015 Regional-scale land-cover change during the 20th century and its consequences for biodiversity. Ambio 44: 17–27. 10.1007/s13280-014-0585-9 PMC4288995 25576277

[r11] Czycholl I, Klingbeil P and Krieter J 2019 Interobserver reliability of the animal welfare indicators welfare assessment protocol for horses. Journal of Equine Veterinary Science 75: 112–121. 31002084 10.1016/j.jevs.2019.02.005

[r12] Dalla Costa E, Dai F, Lebelt D, Scholz P, Barbieri S, Canali E, Zanella AJ and Minero M 2016 Welfare assessment of horses: The AWIN approach. Animal Welfare 25: 481–488.

[r13] Dalla Costa E, Murray L, Dai F, Canali E and Minero M 2014 Equine on-farm welfare assessment: a review of animal-based indicators. Animal Welfare 23: 323–341.

[r14] Debeffe L, McLoughlin PD, Medill SA, Stewart K, Andres D, Shury T, Wagner B, Jenkins E, Gilleard JS and Poissant J 2016 Negative covariance between parasite load and body condition in a population of feral horses. Parasitology 143: 983–997. 27046508 10.1017/S0031182016000408

[r15] Fleurance G, Duncan P, Fritz H, Gordon IJ and Grenier-Loustalot MF 2010 Influence of sward structure on daily intake and foraging behaviour by horses. Animal 4: 480–485. 22443953 10.1017/S1751731109991133

[r16] Fraser D 2003 Assessing animal welfare at the farm and group level: the interplay of science and values. Animal Welfare 12(4): 433–443.

[r17] Fraser D, Weary DM, Pajor EA and Milligan BN 1997 A scientific conception of animal welfare that reflects ethical concerns. Animal Welfare 6(3): 187–205.

[r18] Garrido P, Edenius L, Mikusiński G, Skarin A, Jansson A and Thulin C-G 2021 Experimental rewilding may restore abandoned wood-pastures if policy allows. Ambio 50: 101. 32152907 10.1007/s13280-020-01320-0 PMC7708577

[r19] Garrido P, Mårell A, Öckinger E, Skarin A, Jansson A and Thulin C-G 2019 Experimental rewilding enhances grassland functional composition and pollinator habitat use. Journal of Applied Ecology 56: 946–955.

[r20] Górecka-Bruzda A, Jaworski Z, Jaworska J and Siemieniuch M 2020 Welfare of free-roaming horses: 70 years of experience with Konik Polski breeding in Poland. Animals 10: 1094. 32599935 10.3390/ani10061094 PMC7341202

[r21] Graaf K 2015 Det Gotländska Russets Historia. TGM: Taberg AB, Sweden. [Title translation: The Gotland Pony History]

[r22] Gustavsson E 2007 Grassland plant diversity in relation to historical and current land use. Acta Universitatis Agriculturae Sueciae 106. Department of Ecology, Swedish University of Agricultural Sciences. Uppsala, Sweden.

[r23] Hampson BA, Zabek MA, Pollitt CC and Nock B 2011 Health and behaviour consequences of feral horse relocation. The Rangeland Journal 33: 173–180.

[r24] Harvey AM, Beausoleil NJ, Ramp D and Mellor DJ 2020 A ten-stage protocol for assessing the welfare of individual non-captive wild animals: Free-roaming horses (*Equus ferus caballus*) as an example. Animals 10: 148 31963232 10.3390/ani10010148 PMC7022444

[r25] Hausberger M, Lerch N, Guilbaud E, Stomp M, Grandgeorge M, Henry S and Lesimple C 2020 On-farm welfare assessment of horses: the risks of putting the cart before the horse. Animals 10: 371 32106531 10.3390/ani10030371 PMC7143857

[r26] Henneke D, Potter G, Kreider J and Yeates B 1983 Relationship between condition score, physical measurements and body fat percentage in mares. Equine Veterinary Journal 15: 371–372. 6641685 10.1111/j.2042-3306.1983.tb01826.x

[r27] Hoffmann M 2002 Experiences with grazing in Flemish nature reserves (N. Belgium). Grazing as a conservation management tool in peatland. Report of a Workshop. 22–26 April 2002, Goniadz, Poland. Nature Conservation and Plant Ecology Group, Department of Environmental Sciences, Wageningen University, Wageningen, The Netherlands.

[r28] Kunkle K and Houpt T 2000 Effect of water restriction on equine behaviour and physiology. Equine Veterinary Journal 32: 341–344. 10952384 10.2746/042516400777032200

[r29] Jezierski T, Jaworski Z and Gorecka A 1999 Effects of handling on behaviour and heart rate in Konik horses: comparison of stable and forest reared youngstock. Applied Animal Behaviour Science 62: 1–11.

[r30] Keeling LJ, Rushen J and Duncan IJ 2011 Understanding animal welfare. Animal Welfare 2: 13–26.

[r31] Labartino M, Carnevale G and Thulin C-G 2015 Eco-ethological study on the effect of winter conditions on a population of Gotlandsruss horses and monitoring of their animal welfare. Universita’Degli Studi di Torino, Italy.

[r32] Lesimple C 2020 Indicators of horse welfare: State-of-the-Art. Animals 10: 294 32069888 10.3390/ani10020294 PMC7070675

[r33] Mejdell CM, Bøe KE and Jørgensen GHM 2020 Caring for the horse in a cold climate: Reviewing principles for thermoregulation and horse preferences. Applied Animal Behaviour Science 231: 105071

[r34] Mellor DJ 2016 Updating animal welfare thinking: Moving beyond the ’Five Freedoms’ towards ’A Life Worth Living.’ Animals 6: 21 27102171 10.3390/ani6030021 PMC4810049

[r35] Morel M, McBride S, Chiam R, McKay A, and Ely E 2006 Seasonal variations in physiological and behavioural parameters in a bachelor group of stallion ponies (*Equus caballus*). Animal Science 82(5): 581–590.

[r36] Naundrup PJ and Svenning J-C 2015 A geographic assessment of the global scope for rewilding with wild-living horses (*Equus ferus*). PLoS One 10: e0132359 10.1371/journal.pone.0132359 PMC4503665 26177104

[r37] Piek H 1998 The practical use of grazing in nature reserves in The Netherlands. In: Piek H (ed) Grazing and Conservation Management pp 253–272. Springer: London, UK.

[r38] Popescu S and Diugan E-A 2013 The relationship between behavioural and other welfare indicators of working horses. Journal of Equine Veterinary Science 33: 1–12.

[r39] Research WUL 2012 Welfare monitoring system: assessment protocol for horses. Wageningen UR Livestock Research: Wageningen, The Netherlands.

[r40] Ringmark S, Skarin A and Jansson A 2019 Impact of year-round grazing by horses on pasture nutrient dynamics and the correlation with pasture nutrient content and fecal nutrient composition. Animals 9: 500 31362460 10.3390/ani9080500 PMC6720502

[r41] Saastamoinen M, Herzon I, Särkijärvi S, Schreurs C and Myllymäki M 2017 Horse welfare and natural values on semi-natural and extensive pastures in Finland: Synergies and trade-offs. Land 6: 69

[r42] Sandström J, Bjelke U, Carlberg T and Sundberg S 2015 *Tillstånd och trender för arter och deras livsmiljöer – rödlistade arter i Sverige 2015.* Report no 17. ArtDatabanken, SLU, Uppsala, Sweden. https://res.slu.se/id/publ/67065. [Title translation: Conditions and trends of species and their habitats - the Swedish red list 2015]

[r43] Scott DW and Miller WH 2010 Equine Dermatology-E-Book. Elsevier Health Sciences: London, UK.

[r44] Sherwin CM, Christiansen SB, Duncan IJ, Erhard HW, Lay Jr DC, Mench JA, O’Connor CE and Petherick JC 2003 Guidelines for the ethical use of animals in applied ethology studies. Applied Animal Behaviour Science 81: 291–305.

[r45] Slivinska K and Kopij G 2011 Diet of the Przewalski’s horse (*Equus przewalskii*) in the Chernobyl exclusion zone. Polish Journal of Ecology 59: 841–847. www.smhi.se

[r46] Swedish Board of Agriculture (SBA) 2016 *Naturbetesmarker - en resurs i vår hästhållning. Jordbruksinformation 9-2016.* www.jordbruksverket.se. [Title translation: Natural pastures – a resource in horse husbandry]

[r47] Swedish Board of Agriculture (SBA) 2018a *Djurskyddslag. SJVFS 2018:* 1192. www.jordbruksverket.se. [Title translation: The Swedish Animal Welfare Act]

[r48] Swedish Board of Agriculture (SBA) 2018b *Föreskrifter om ändring i Statens jordbruksverks föreskrifter och allmänna råd (SJVFS 2015:25) om miljöersättningar, ersättningar för ekologisk produktion, kompensationsstöd och djurvälfärdsersättningar.* www.jordbruksverket.se. [Title translation: Regulations on alterations in the Swedish Board of Agricultures regulations and general recommendations related to environmental-, ecological production- and animal welfare compensations]

[r49] Swedish Board of Agriculture (SBA) 2019a Animal welfare ordinance. *SJVFS* 2019*:*66. Sweden www.jordbruksverket.se

[r50] Swedish Board of Agriculture (SBA) 2019b *Statens jordbruksverks föreskrifter och allmänna råd om hästhållning - Saknr L 101.* *SJVFS* 2019*:*17 *L 101.* www.jordbruksverket.se. [Title translation: Swedish Board of Agricultures Guidelines and General Recommendations on Horse Husbandry]

[r51] Swedish Meteorological and Hydrological Institute (SMHI) 2021 *Weather reports.* www.smhi.se

[r52] Tydén E, Jansson A and Ringmark S 2019 Parasites in horses kept in a 2.5 year-round grazing system in nordic conditions without supplementary feeding. Animals 9: 1156 31861066 10.3390/ani9121156 PMC6940839

[r53] Tälle M, Deák B, Poschlod P, Valkó O, Westerberg L and Milberg P 2016 Grazing vs mowing: A meta-analysis of biodiversity benefits for grassland management. Agriculture, Ecosystems & Environment 222: 200–212.

[r54] Viksten S, Visser E, Nyman S and Blokhuis H 2017 Developing a horse welfare assessment protocol. Animal Welfare 26: 59–65.

[r55] Viksten SM 2016 Improving horse welfare through assessment and feedback. Department of Animal Environment and Health, p 93. Swedish University of Agricultural Sciences: Uppsala, Sweden.

[r56] Visser E, Neijenhuis F, de Graaf-Roelfsema E, Wesselink H, De Boer J, van Wijhe-Kiezebrink M, Engel B and Van Reenen C 2014 Risk factors associated with health disorders in sport and leisure horses in the Netherlands. Journal of Animal Science 92: 844–855. 24352963 10.2527/jas.2013-6692

[r57] Visser EK and Van Wijk-Jansen EE 2012 Diversity in horse enthusiasts with respect to horse welfare: An explorative study. Journal of Veterinary Behavior 7: 295–304.

[r58] Wright B, Rietveld G and Lawlis P 1998 Body Condition Scoring of Horses - Factsheet. Canadian Ministry of Agriculture, Food and Rural affairs, 98-101 (AGDEX 460/28). Ontario, Canada. http://www.omafra.gov.on.ca/english/livestock/horses/facts/98-101.htm

